# Supplementary figures and images for: Novel epigenetic clock for fetal brain development predicts prenatal age for cellular stem cell models and derived neurons
Source: Mol Brain. 2021 Jun 26;14:98. doi: 10.1186/s13041-021-00810-w (PMC8236187; doi:10.1186/s13041-021-00810-w)

**A Training Data**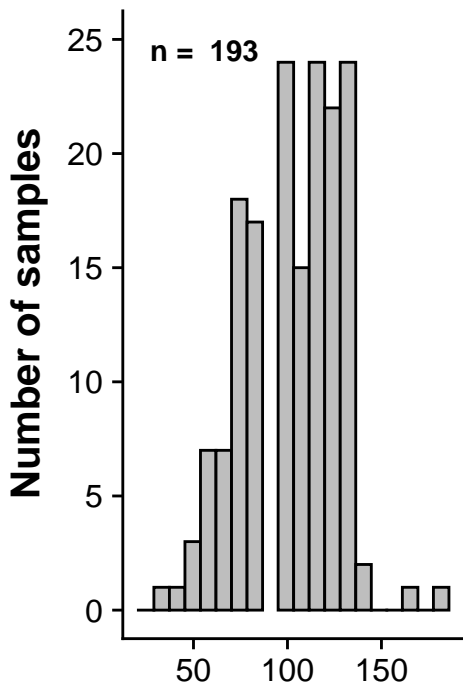**B Testing Data**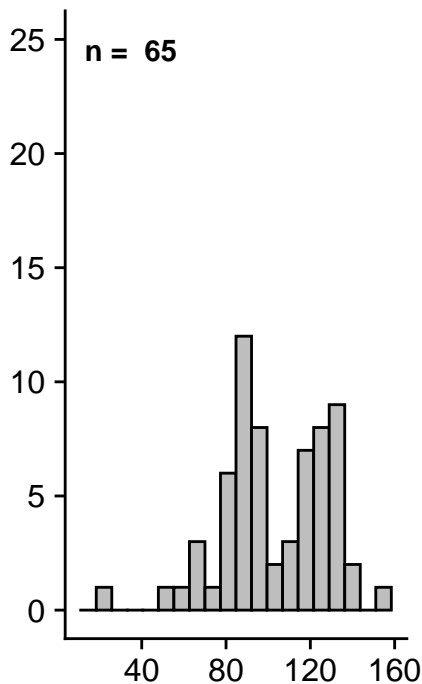**C Validation Data**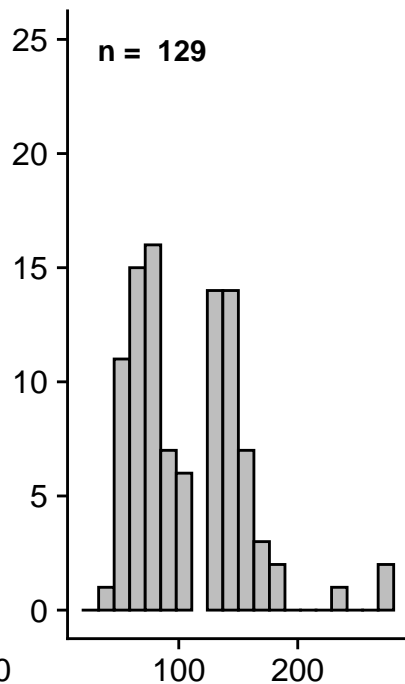

**Chronological Age**  
*in days post-conception*

Supplement: Supplementary file 3 — Additional file 3: Fig. S1. Histogram of age distribution. Chronological age of the fetal samples measured in days post-conception in A) training data, B) testing data and C) validation data. [file 13041_2021_810_MOESM3_ESM.pdf]

**A FBC**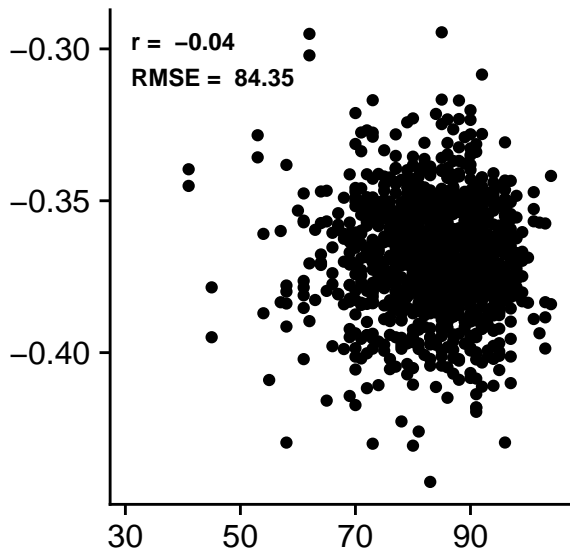**B MTC**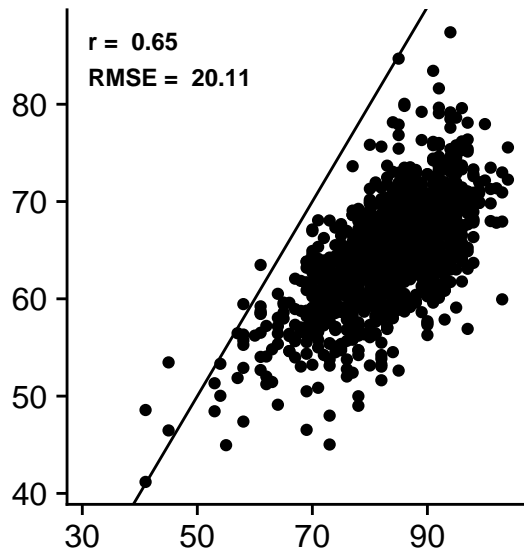**C GAC**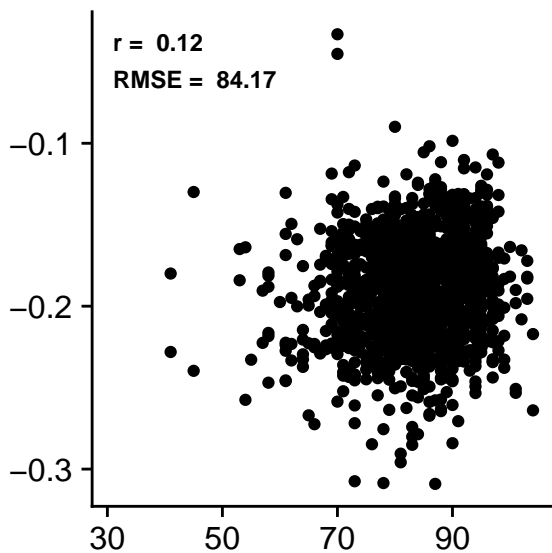**D CPC**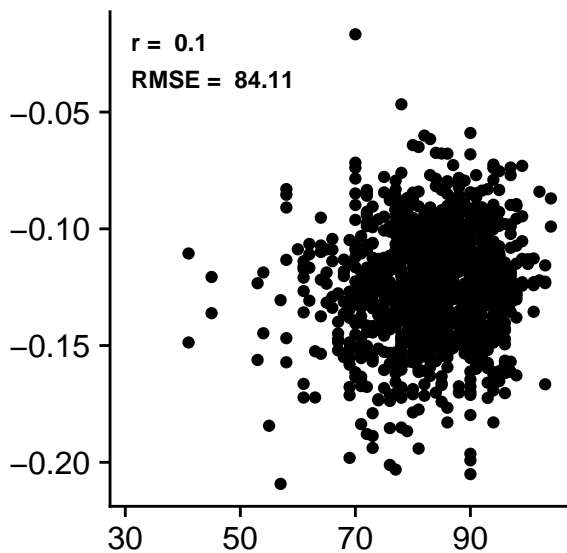

**Predicted Age**  
*in years*

**Chronological Age**  
*in years*

Supplement: Supplementary file 5 — Additional file 5: Fig. S3. Comparison of predictions from the four DNAm clocks in adult brain samples (n = 1221). Shown are scatterplots comparing chronological age (x-axis; years) against predicted epigenetic age (y-axis; years) calculated using A Fetal Brain Clock (FBC); B Horvath’s Multi Tissue Clock (MTC); C Knight’s Gestational Age Clock (GAC); D Lee’s Control Placental Clock (CPC) in an independent adult brain dataset. Where necessary, predicted age was converted to years, where 0 indicates birth. The black line indicates the identity line of chronological and predicted epigenetic age and represents a perfect prediction. Two statistics were calculated to evaluate the precision of each DNAm clock: Pearson’s correlation coefficient (r) and the root mean squared error (RMSE). [file 13041_2021_810_MOESM5_ESM.pdf]
