# Supplementary material for: Novel epigenetic clock for fetal brain development predicts prenatal age for cellular stem cell models and derived neurons
Source: Mol Brain. 2021 Jun 26;14:98. doi: 10.1186/s13041-021-00810-w (PMC8236187; doi:10.1186/s13041-021-00810-w)
Supplement: Supplementary file 4 — Additional file 4: Fig. S2. Comparison of predictions from the four DNAm clocks in the training data (n = 193). Shown are scatterplots comparing chronological age (x-axis; days post-conception) against predicted epigenetic age (y-axis; days post-conception) for A Fetal Brain Clock (FBC); B Horvath’s Multi Tissue Clock (MTC); C Knight’s Gestational Age Clock (GAC); D Lee’s Control Placental Clock (CPC) in the data used for training of the FBC. Where necessary, predicted age was converted to days post-conception. The black line indicates the identity line of chronological and predicted epigenetic age and represents a perfect prediction. Two statistics were calculated to evaluate the precision of each DNAm clock: Pearson’s correlation coefficient (r) and the root mean squared error (RMSE). [file 13041_2021_810_MOESM4_ESM.pdf]

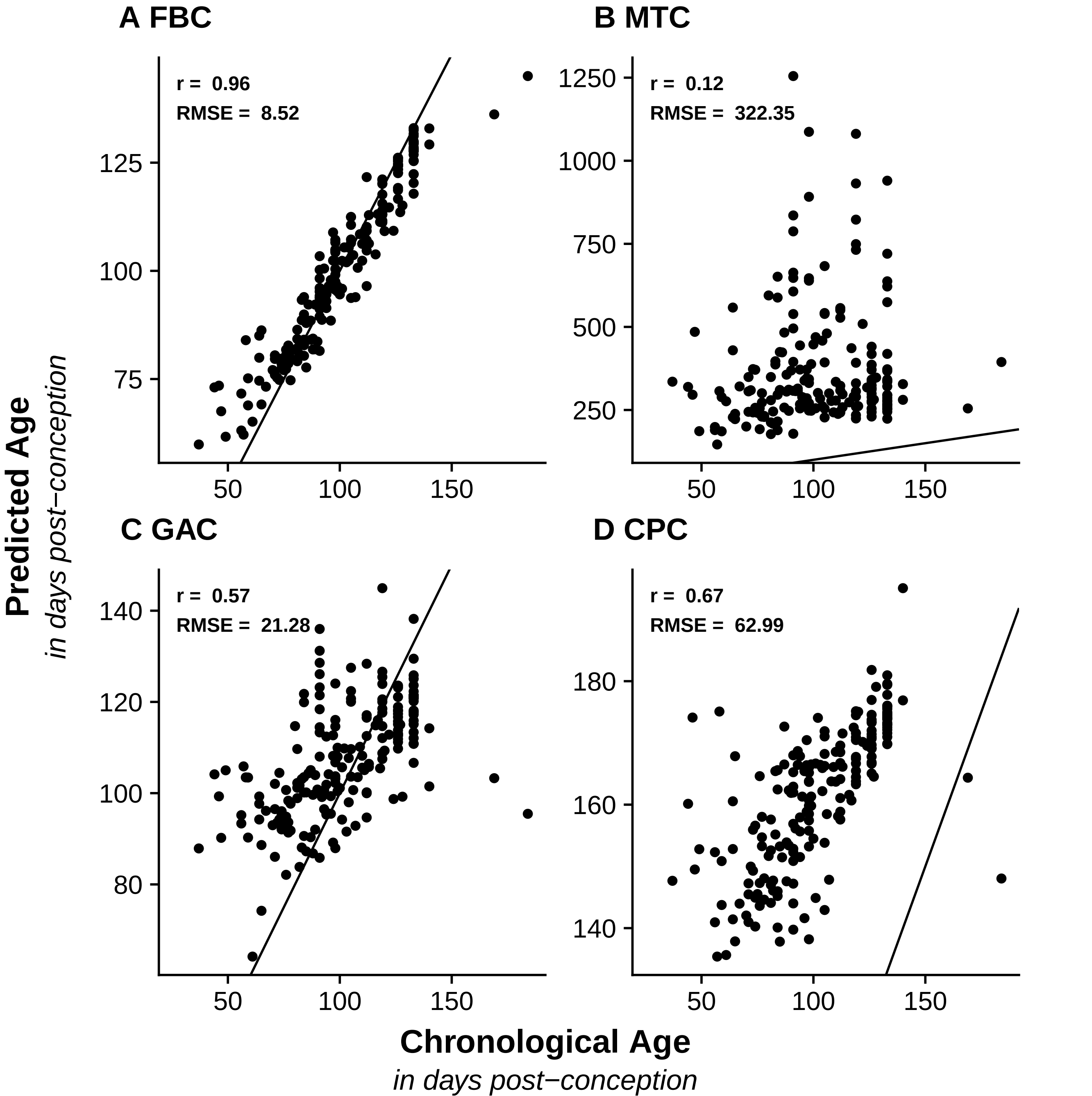
A FBC
r = 0.96
RMSE = 8.52
B MTC
r = 0.12
RMSE = 322.35
C GAC
r = 0.57
RMSE = 21.28
D CPC
r = 0.67
RMSE = 62.99
Predicted Age
in days post−conception
Chronological Age
in days post−conception
50
100
150
75
125
250
500
750
1000
1250
80
120
140
160
180
